# A novel compound heterozygous of β-thalassemia with HbG-Coushatta: case report of Iran

**DOI:** 10.1038/s41439-023-00243-y

**Published:** 2023-05-15

**Authors:** Narges Soozangar, Ehsan Abbaspour, Haleh Mokaber, Zahra Nematollahi, Behzad Davarnia

**Affiliations:** 1 Department of Medical Genetics and Pathology, Ardabil University of Medical Sciences, Ardabil, Iran; 2 Zoonoses Research Center, Ardabil University of Medical Sciences, Ardabil, Iran

**Keywords:** DNA sequencing, Genetics research

## Abstract

A 30-year-old male couple from Ardabil city, Iran, were admitted for premarital screening. An abnormal band in HbS/D regions with high levels of HbF and HbA 2 led us to suspect the possibility of a compound heterozygous state of β-thalassemia in our affected proband. Therefore, beta globin chain sequencing of proband discovered a heterozygote combination of the Hb G-Coushatta [b22 (B4) Glu>Ala, HBB: c.68A>C) with HBB: IVS-II-1 (G>A) mutation as a compound heterozygote.

## Case report

Hereditary hemoglobinopathies, including quantitative abnormalities (thalassemia) and qualitative abnormalities (structural hemoglobin variants), cause abnormal Hb structure^1^. Beta (β)-thalassemia is known as an inherited blood disorder characterized by an absent (β^0^) or reduced (β^+^) synthesis of the β-globin subunit of hemoglobin (Hb) in common homozygous autosomal recessive (AR) patterns^2^.

Hemoglobin G-Coushatta with the preferred name NM_000518.4(HBB):c.68A>C (p.Glu23Ala) is an abnormal β-chain structural Hb caused by the substitution of the glutamyl group within residue at position 22 [β22 (B4)Glu →Ala]^3^. This type of Hb variant was identified in various countries, such as Japan, Algeria, Korea, Turkey, and Thailand^3–7^.

Considering that different ethnic groups live in our country, there are lots of various types of heterogeneous clinical and genetic characteristics of hemoglobinopathies in Iran^8,9^. The following report aimed to describe an uncommon compound heterozygous state of β-thalassemia trait with HbG-Coushatta for the first time in Iran.

**Fig. 1:**
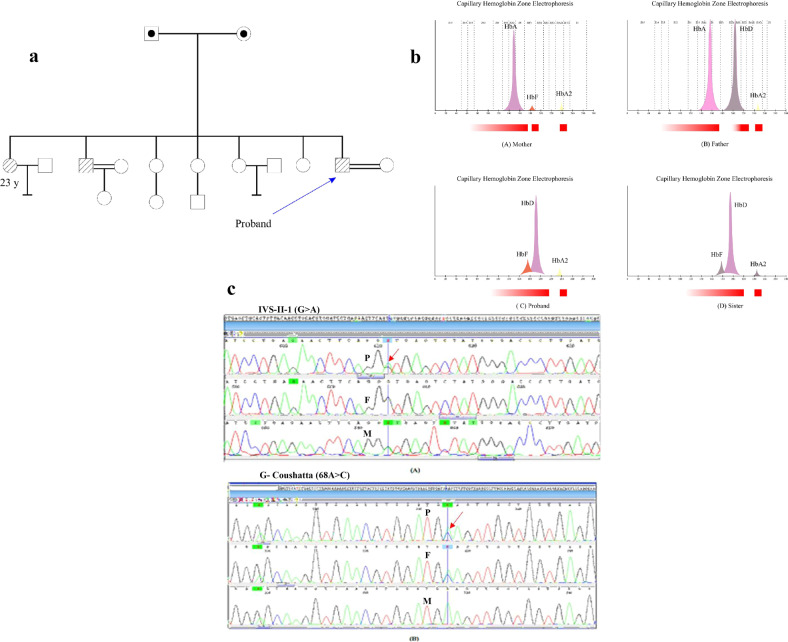
Pedigree and HPLC electrophoresis results and Hb variants sequencing analysis of patients with HbG-Coushatta. **a** Familial pedigree. The proband is marked with the arrow. **b** Pattern of Hb analysis by gel electrophoresis: Mother (A) with β-thalassemia carrier pattern. (B) proband (C) and his sister (D) with β-thalassemia carrier pattern and abnormal Hb indices between zones 5 and 6. **c** DNA sequencing electropherogram: The top part shows a common heterozygote mutation of HBB: c.IVS II-1 G>A for proband and his mother and the below indicates a c.68A>C, HbG-Coushatta for proband and his father, which is marked in a vertical line.

A couple, including a 30-year-old man and a 25-year-old woman from Ardabil city, Iran, were referred to the Reference Laboratory of Health Center (RLHC) for premarital screening. A familial pedigree was carefully drawn (Fig. 1a).

Venous peripheral blood was collected and each participant was subject to CBC (complete blood count) analysis and Hb-electrophoresis. The woman had normal CBC and Hb electrophoresis, but hematological features of the man showed microcytic hypochromic anemia (Hb 10.6 g/dl) with low MCH (19 pg) and MCV (54.7 fl). Hb electrophoresis analysis results revealed an increasing level of HbF (11.8%) and HbA_2_ (5.8%) (Table 1). According to the findings, an abnormal band was also identified within HbS/D zone between HbA and HbA_2_ (Fig. 1b). An abnormal band in HbS/D regions with high HbF and HbA_2_ levels led us to suspect the possibility of a compound heterozygous state of β-thalassemia in our affected proband.

**Table 1:** The hematological parameters.

Parameters	Proband	Father	Mother	Sister
*Complete blood count*				
WBC (10^3^/ μl)	6.12	6.0	9.3	5.8
RBC (10^6^/μL)	5.59	5.2	5.83	5.4
Hb(g/dL)	10.6	15.3	11.2	10.2
HCT (%)	30.6	43.3	38	30
MCV (fL)	54.7	83.7	65	52.4
MCH (pg)	19	29.4	19.2	20
MCHC(%)	34.6	35.2	29.5	33.2
PLT (10^3^/μL)	368	241	284	292
*Fractions of Hb variants by HPLC*				
Hb A (%)	_	54.6	90.8	_
Hb A_2_ (%)	5.8	2.9	5.1	5.1
Hb F (%)	11.8	_	4.1	10.6
Hb D (%)	82.4	42.5	_	84.3

*Hb* Hemoglobin, *HCT* hematocrit, *HPLC* high-performance liquid chromatography, *MCH* mean cell hemoglobin, *MCHC* mean cell hemoglobin concentration, *MCV* mean cell volume, *PLT* Platelet, *RBC* red blood cells, *WBC* white blood cells.

Molecular genetic tests study β-globin chain variations to determine individuals who are at risk^2^. DNA was extracted from the selected whole blood samples. Direct gene sequencing was subsequently performed to identify proband, parents, and his siblings of β-globin gene changes.

The collected data of the hematological parameters and Hb gel electrophoresis of the parents and related sibling are described respectively in Table 1. According to hematological features, the proband showed an anisocytosis and microcytic hypochromatic anemia (Hb=10.6 g/dL) with low MCH (19 pg) and MCV (54.7 fl). In addition, his Hb zone electrophoresis had a significant increase in the level of HbF (11.8 %), HbA_2_ (5.8%), and HbD (82.4%). Analyzing the mother’s sample electrophoresis identified a β-thalassemia carrier pattern with a decreased level of HbA1 (90.8%) while increased HbF (4.1%) and HbA_2_ (5.1%) levels. Compared to the mother, the father’s results are as follows: low level of HbA_1_ (54.6%), a high level of HbD (42.5%) and a normal HbA_2_ level. Considering the results of siblings, there is a consistency with proband as his sister’s blood electrophoresis analysis showed a high HbF (10.6%), high HbA_2_ (5.1%), and high HbD (84.3%) levels (Fig. 1b).

In respect of the hematological data, there was a β-thalassemia carrier condition in proband and his mother, while an abnormal structural hemoglobinopathy of HbS/HbD was indicated in proband, his father, and sister. Thus, the genetic diagnostic testing of β-globin gene (HBB) has to preferably be performed in interfamilial individuals to detect the precise genetic cause of the disease. As a result, β-globin chain sequencing of proband discovered a heterozygote combination of the HbG-Coushatta [b22 (B4) Glu>Ala, HBB: c.68A>C) with HBB: IVS-II-1 (G>A) mutation as a compound heterozygote, which have been inherited from both parents in an autosomal recessive pattern (Fig. 1c). In the following, sequencing analysis of HBB gene in the father revealed a heterozygote point mutation at codon 68 (G*A*A→G*C*A), which is known as HbG-Coushatta [β22 (B4) Glu>Ala, HBB: c.68A>C) (Fig. 1c). While the mother sequencing revealed a common mutation at IVS-II-1 (G>A) nucleotide position, which reduced β-globin chain products to the extent that a heterozygous condition requires.

The prevalence of HbG-Coushatta appears to be quite rare; it has been reported higher than expected rate. One of the critical reasons for low reporting of this abnormal Hb might be the inability to separate HbG-Coushatta with HbQ and HbD in electrophoretic mobility^10^. However, in this study after performing electrophoresis, we became suspicious type of the hemoglobinopathies owing to the abnormal Hb types in HbS/HbD results. Considering that the abnormal HbS could not be discriminated with other proteins present in serum through capillary electrophoresis for that reason these variants moving slowly in alkaline gel, these variants are ultimately recognized by DNA sequencing, especially when they have unexpected hematological changes.

HbG-Coushatta has the substitution of the negatively charged glutamyl group with a neutral alanine residue at position 22^3^. This replacement typically affects the distribution of charges and impairs molecular function. Nevertheless, this replacement at position 22 of the β-globin chain is not associated with heme chains and is expected to be hematologically normal. Here, our proband and his father with heterozygous HbG-Coushatta were also clinically silent, as we expected.

Molecular identification of an HbG-Coushatta case report in Malaysia reported a 33-year-old male who was asymptomatic. He also had an increased red blood cell (RBC) count (5.91×10^6^g/dl), normal Hb (16.09 g/L), and RBCs appeared hypochromic microcytic. His laboratory test results showed a decreased HbA (45.6%) and normal HbF (0.3%) levels. In the screening method, β-globin gene sequencing revealed a heterozygous mutation of HbG-Coushatta [β22 (B4) (GAA→GCA)]^11^. The other case was a mother of a 5-month-old infant from Thailand diagnosed with compound heterozygosity for HbE (*G*AG→*A*AG) and HbG-Coushatta. The case had abnormal HbX (50.3%), HbA2/E (46.7%), HbA (0%), and HbF (3%) values^4^.

A recent study of a Turkish family revealed the homozygous variant of HbG-Coushatta in a father, heterozygous variant of HbD Punjab in a mother and heterozygous HbG-Coushatta in their baby. Both of homozygous father and heterozygous baby for HbG-Coushatta had normal HbA2 (3.2% and 3.1%, respectively) and normal HbF (0% and 2.4%, respectively) and a great decrease in HbA (0% and 55.4%, respectively). However, there is no hematological or clinical symptom^12^. The next case was a Sri Lankan girl whose brother was β-thal trait. The CBC results indicated a noticeable decrease in MCV, MCH, MCHC and Hb values in proband, her brother, and mother, respectively. Analysis of this family’s Hb showed an abnormal HbA2/E in both father and proband. Increased levels of HbA2 in proband’s brother and mother were similar to the β-thal trait. There is a normal HbF in all members of the family. Consequently, β-globin sequencing showed that the proband has a compound heterozygous of HbG-Coushatta with β-thal mutation, IVS-I-5^13^.

Obviously, a limited number of cases have been reported regarding this variant, either in homozygous or heterozygous form. It was observed that the levels of HbF and HbA2 were normal, but HbA in the homozygous state was much lower than in the heterozygous state (0 and 45.6%, respectively). The cases of compound heterozygous of HbG-Coushatta with HbE or HbD showed a slight increase in HbF, normal HbA2 and increased HbA. In our report, we have seen a very pointed decrease in HbA (0%), which was similar to the case of a compound heterozygous of HbG-Coushatta with IVS-I-5 β-thal mutation (HbA=4%). This severe decrease in HbA may be due to the combination of HbG-Coushatta with a β-thal mutation. On the other hand, we had a sharp increase in HbF (11.8), which was not observed in the mentioned cases.

In the report of a compound heterozygous of HbG-Coushatta with a β-thal mutation IVS-I-5 has been reported a normal HbF. This is different compared to our finding and the difference may be due to the type of β-thalassemia mutation. This is the first report of a compound heterozygous state of a common β-thalassemia mutation IVS-II-1 with HbG-Coushatta as a combination of the one quantitative with qualitative alleles. There are no clinical symptoms or signs related to the compound heterozygous of HbG-Coushatta with a β-thal mutation while having an increase in HbF and a decrease in HbA levels. However, the frequency and clinical data of the HbG-Coushatta are to be completely very rare around the world. It is noteworthy that electrophoretic mobility of HbG-Coushatta is similar to other hemoglobins such as HbS and HbD. Therefore, in such cases, family studies and molecular genetic testing are recommended.

## HGV Database

The relevant data from this Data Report are hosted at the Human Genome Variation Database at 10.6084/m9.figshare.hgv.3295, 10.6084/m9.figshare.hgv.3301.
